# An Experience in a Special Hospital

**Published:** 1913-11-08

**Authors:** 


### HOSPITALS FROM THE PATIENTS' POINT OF VIEW.

(Contributions to this Section are Invited.)

### An Experience in a Special Hospital.

The patient who hereunder gives her experience was in a general ward of a special hospital, and had been operated upon first of all "in private." On account of certain complications she was transferred to hospital. The operation was the removal of the nail of the toe; and the patient at no time during her stay was in a serious condition, so she had the opportunity of keen observation and plenty of time "on hand." She says: I had always had a great dread of hospitals and would never hear of going into one, but after much persuasion was prevailed upon to do so. I was put in a ward where there were eighteen beds, whose occupants' ages ranged from a little over fourteen years to my own (middle age). I was very much struck by the freedom that was allowed the patients in the ward; they were given every opportunity to talk and laugh, and, indeed, on some occasions we might have been heard singing and sometimes we played games (at the time there were no critical cases in the ward); but there was good discipline; the patients seemed to know that they had to do as they were told and readily "obeyed orders."

The sister of the ward was "a dear"; she would do anything for any of us and would never refuse anything that would make us happy if it was reasonable, and the nurses were kindness itself and did everything they could for me. I remember one night I had just gone to sleep when one of the nurses came to dress my foot; she woke me up so gently and kindly. I did not sleep the first three nights I was in the hospital because everything I suppose was strange, and there was a lot of noise which came from passing traffic as we were very near the road and the windows opened in that direction; but the night nurses were very kind and attentive, and once during the night the doctor came round the wards.

On Sundays we had a very nice service, which everyone seemed to enjoy; we chose the hymns beforehand and an organist came in to play them, and every night someone or other of us would choose a hymn which we would all sing before "lights out."

The food was very plain but very good, and much better than I had expected it to be, and I can only say that through all the time I was in the hospital *everybody* was very kind and thoughtful, and that my fear and dread of hospitals has quite gone; and when I was asked the other day by a friend "what the hospital was like, because so many people grumbled at hospitals," I said that I had nothing to grumble about, and should always think of the kindness that had been shown me while I was a patient.

